# Characteristics and outcomes of men with erectile dysfunction as the presenting symptom due to a lactotroph adenoma

**DOI:** 10.1007/s00701-024-06213-9

**Published:** 2024-07-31

**Authors:** Lukas Andereggen, Angelo Tortora, Gerrit A. Schubert, Christian Musahl, Janine Frey, Andrea Stieger, Béatrice Kobel, Markus M. Luedi, Michel Roethlisberger, Luigi Mariani, Jürgen Beck, Emanuel Christ

**Affiliations:** 1 Department of Neurosurgery, Kantonsspital Aarau, Aarau, Switzerland; 2 Faculty of Medicine, University of Bern, Bern, Switzerland; 3 Deptartment of Neurosurgery, RWTH Aachen University, Aachen, Germany; 4 Department of Gynecology and Obstetrics, Luzerner Kantonsspital, Lucerne, Switzerland; 5 Department for Anesthesiology, Intensive, Rescue and Pain Medicine, Kantonsspital St.Gallen, St.Gallen, Switzerland; 6 Department of Anaesthesiology and Pain Medicine, Inselspital, Bern University Hospital, University of Bern, Bern, Switzerland; 7 Department of Neurosurgery, University Hospital of Basel, Basel, Switzerland; 8 Faculty of Medicine, University of Basel, Basel, Switzerland; 9 Department of Neurosurgery, Medical Center, University of Freiburg, Freiburg, Germany; 10 Department of Neurosurgery, Inselspital, Bern University Hospital, University of Bern, Bern, Switzerland; 11 Department of Endocrinology, Diabetes and Metabolism, University Hospital of Basel, Basel, Switzerland

**Keywords:** Erectile dysfunction, Dopamine agonist, Prolactinoma, Transsphenoidal surgery

## Abstract

**Purpose:**

Erectile dysfunction (ED) is frequently underreported in men suffering from prolactinomas and can be challenging to manage. Both dopamine agonists (DAs) and transsphenoidal surgery (TSS) correct hyperprolactinemia and restore gonadal function. However, there is scarce data regarding their effectiveness in correcting ED over the long term.

**Methods:**

This study is a retrospective single-center comparative cohort study analyzing men diagnosed with prolactinomas, both with and without confirmed erectile dysfunction (ED) at diagnosis. Independent risk factors for persistent ED over the long term were examined using multivariate logistic regression.

**Results:**

Among the 39 men with lactotroph adenomas, ED was one of the presenting symptoms in 22 (56%). The mean age at diagnosis was 45 ± 12 years. Surgery was the primary treatment in 6 (27%) ED patients and 8 (47%) non-ED patients. After a mean follow-up of 74 ± 48 months, remission from hyperprolactinemia was achieved in the majority (76%) of men: 71% in the non-ED cohort and 81% in the ED group (*p* = 0.70), regardless of the primary treatment strategy (surgical 84% versus medical 72%, *p* = 0.46). Long-term remission of ED was noted in 16 (73%) patients. Interestingly, high baseline BMI levels emerged as potential risk factors for persistent ED over the long term (OR 1.4, 95%CI 1.0–1.9; *p* = 0.04), while neither the initial adenoma size nor the primary treatment strategy (i.e., TSS vs. DAs) reached statistical significance.

**Conclusions:**

Correcting hyperprolactinemia and its associated hypogonadism significantly improves ED in the majority of men with prolactinomas over the long term, regardless of the primary treatment strategy employed. In addition to addressing endocrine deficiencies, the early initiation of weight control programs may be considered for men with lactotroph adenomas and ED. Although our study suggests an association between BMI and the risk of persistent ED, further research is needed to establish any causal relationships.

## Introduction

Hyperprolactinemia and associated hypogonadism are rare causes of erectile dysfunction (ED) in men, with serum testosterone levels being measured within the normal range in about half of them [16, 28, 43]. Notably, improvement in ED tends to correlate more with the resolution of hyperprolactinemia than with an increase in testosterone levels [23, 27]. Both dopamine agonists (DAs) and transsphenoidal surgery (TSS) correct hyperprolactinemia and restore gonadal function in men with prolactinoma[2, 4–7, 10–12, 26, 35, 36], yet data on their effectiveness in improving ED over the long term are scarce. In this study, we aimed to compare the characteristics and long-term outcomes of men presenting with and without ED as the initial symptom related to an underlying prolactinoma, and to determine predictors associated with persistent ED.

## Methods

### Study design

We conducted a retrospective comparative study using data from a prospectively maintained institutional database spanning from January 1996 to December 2015. We analyzed men with prolactinomas, whether presenting with or without erectile dysfunction (ED). ED was defined as the failure to achieve or maintain a rigid penile erection suitable for sexual intercourse, as assessed by the International Index of Erectile Function (IIEF-5)[51] score of ≤ 7. Clinical and biochemical characteristics, as well as outcome parameters, were compared between both cohorts. All patients met the diagnostic criteria for a prolactin (PRL)-secreting pituitary adenoma. The Human Research Ethics Committee of Bern (Kantonale Ethikkommision KEK Bern, Bern, Switzerland) approved the study (KEK no. 10–10-2006 and 8–11-2006).

### Biochemical assessment

Anterior pituitary gland hormone and prolactin (PRL) levels were assessed. For the latter, the immunoradiometric PRL assay was utilized, employing serum dilution to overcome the high-dose PRL hook effect [38]. The upper limit of PRL levels was set at 20 µg/L [44]. Partial hypopituitarism was defined as impaired secretion of one or more pituitary hormones. Low serum cortisol levels (< 50 nmol/L) or normal cortisol levels but inadequate responses to the adrenocorticotropin (ACTH) stimulation test or insulin tolerance test indicated secondary adrenal insufficiency. Secondary hypothyroidism was diagnosed when there were low to normal thyroid-stimulating hormone (TSH) levels and low free thyroxin (FT4) levels. A gonadotropin deficiency or central hypogonadism was considered in cases of low to normal levels of gonadotropins alongside low total testosterone levels. Immunohistochemical analysis, performed according to the WHO classification for neuroendocrine tumors, was confirmed in the surgical cohort[53].

### Assessment of cardiovascular risk factors and body mass index (BMI)

Cardiovascular risk factors [24, 25, 42] (i.e., hypertension, diabetes, smoking), and standard BMI[41] was assessed for all patients, with a BMI of 19–25 kg/m^2^ considered normal.

### Radiological assessment

Dynamic MRI-sequences of the sellar region were performed using a standardized protocol including a proton density/T2-weighted whole-brain scan with 5 mm slice thickness and both unenhanced and contrast-enhanced overlapping 3 mm scans in the sagittal and coronal planes over the sellar region, as previously reported. A diameter of 1–10 mm was indicative of a microadenoma and > 10 mm as a macroadenoma. The Knosp classification was used to define the infiltration of the cavernous sinus by the prolactinoma[39, 45].

### Prolactinoma treatment

Prolactinoma treatment consisted of either dopamine agonists (DAs) or transsphenoidal surgery (TSS). The preferred treatment was determined on a case-by-case basis at the discretion of a weekly interdisciplinary pituitary specialist board. In addition to visual impairments associated with cystic tumor configuration, the decision to pursue TSS was also influenced by patients' preference for surgery over long-term DA-agonist therapy [34]. It is worth noting that health insurance in Switzerland covers both medical and surgical treatments as primary options for lactotroph adenomas, thus eliminating selection and cost biases in treatment decision-making. Pituitary surgery was conducted using a transseptal, transsphenoidal microsurgical approach (TSS) with sellar reconstruction as described before [8, 9, 13].

### Long-term assessment

At the last follow-up, the presence of persistent ED (i.e., severe ED, defined as IIEF-5 ≤ 7) versus non-ED was assessed. Regarding PRL levels, dopamine agonists (DAs) were tapered 24 months after the initiation of medical therapy in case of normalization [22, 57]. Recurrence was defined as an increase in PRL levels above the normal range (> 20 µg/L) during the last follow-up period after a previous remission, regardless of radiological findings [48, 49].

### Data availability statement

The data supporting the findings of this study are available upon request.

### Statistical analysis

Data were analyzed using IBM SPSS statistical software (V29.0 Software, IBM Corp., New York, NY, USA), and GraphPad Prism (V9.0 software, San Diego, CA, USA). Continuous variables were examined for homogeneity of variance and are expressed as mean ± SD unless otherwise noted. Serum PRL levels are presented as median values and interquartile range (IQR). Categorical variables are given as numbers and percentages. For comparisons of means between groups (i.e., in patients with versus without ED), Student’s t-test was used for normally distributed data, and the Mann–Whitney test for nonparametric data. The Wilcoxon signed-rank test was used to evaluate paired differences in PRL, testosterone, and BMI levels before and after treatment. Categorical variables were compared using Pearson’s chi-square test or Fisher’s exact test, as appropriate.

We assessed the proportion of men with long-term persistence of ED and performed time-dependent multivariable regression analysis to calculate hazard ratios (HR) for potential risk factors. The variables tested were age at diagnosis, headache at presentation, hypogonadism at diagnosis, BMI (kg/m^2^), initial PRL and testosterone levels, adenoma size and invasion, and the primary treatment approach. The multivariable regression analysis included all dependent risk factors in the univariable regression with a p-value ≤ 0.3. The Spearman rank-order correlation coefficient was calculated to check for the strength of association between PRL values and patients’ BMI. Baseline PRL values were log-transformed before being imputed in both the correlation and the regression analysis, as the data showed a positively skewed distribution. The significance level was set at *p* = 0.05.

## Results

### Baseline characteristics

**Fig. 1:**
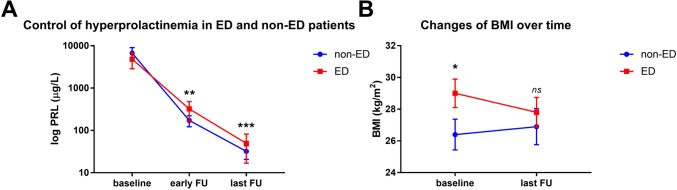
Changes in PRL and BMI levels over time (**A**) PRL levels were not significantly different at baseline when comparing both cohorts (*p* = 0.55). They significantly decreased over the long term; namely, in the non-ED cohort from 2628 µg/L (IQR 694–9159 µg/L) to 16 µg/L (IQR 9–30 µg/L, *p* = 0.01), and in the ED cohort from 1831 µg/L (IQR 768–4457 µg/L) to 13 µg/L (IQR 5–24 µg/L, *p* = 0.03), respectively. At last follow-up, serum PRL levels were not different between the two groups (*p* = 0.65). (**B**) Men with ED presented with a significantly higher body mass index (BMI, *p* = 0.01), but no significant changes in BMI were noted over the long term in ED or non-ED patients. At last follow-up, BMI values between the two cohorts were not significantly different (*p* = 0.17)

Patient characteristics at diagnosis are summarized in Table 1. Of the 39 men with lactotroph adenomas meeting study inclusion criteria, ED was one of the presenting symptoms noted in 22 (56%) of them. Surgery was the primary treatment in 6 (27%) ED patients and 8 (47%) non-ED patients, respectively (*p* = 0.31). The primary treatment strategy (i.e., TSS or DAs), patient age, cardiovascular risk factors, baseline PRL levels, and the prevalence of macroprolactinoma or adenomas with cavernous sinus invasion were not significantly different between patients with and without ED. Compared to those without erectile dysfunction, men experiencing ED presented with a significantly higher body mass index (BMI 29 ± 4.2 kg/m^2^ vs. 26.4 ± 4 kg/m^2^, *p* = 0.01; Fig. 1A), decreased testosterone concentrations (3.1 ± 2.1 nmol/L vs. 7.4 ± 3.6 nmol/L, *p* = 0.04), and higher rates of hypogonadism (96% vs. 65%, *p* = 0.03).

**Table 1:** Patient characteristics at baseline

Characteristics at diagnosis	Non-ED	ED	All patients	P-value
Number of patients, n (%)	17 (44%)	22 (56%)	39 (100%)	
Age (years), mean ± SD	42 ± 15	48 ± 10	45 ± 12	0.14
BMI (kg/m2), mean ± SD	26.4 ± 4.0	29 ± 4.2	28.4 ± 4.5	0.01
PRL levels (ug/L), median (IQR)	2628 (694–9159)	1831 (768–4457)	1979 (768–5039)	0.55
Affected pituitary axes, n (%)				
Secondary adrenal insufficiency	0 (0)	2 (9)	2 (5)	0.5
Secondary hypothyroidism	0 (0)	3 (14)	3 (8)	0.24
Gonadotropin deficiency	11 (65)	21 (96)	32 (82)	0.03
LH (IU/L), mean ± SD	1.8 ± 0.9	1.3 ± 0.8	1.6 ± 0.8	0.16
FSH (IU/L), mean ± SD	2.9 ± 1.5	2.2 ± 1.1	2.5 ± 1.6	0.11
Testosterone levels (nmol/L), mean ± SD	6.7 ± 4.9	6.2 ± 4.8	6.2 ± 4.7	0.72
Adenoma size (i.e. macroadenoma)	12 (71)	17 (77)	29 (74)	0.72
Cavernous sinus invasion, n (%)	10 (59)	13 (59)	23 (59)	0.99
Vascular risk factors, n (%)	10 (67)	15 (79)	25 (74)	0.46
Smoking	3 (21)	3 (16)	6 (18)	0.99
Diabetes	1 (7)	4 (21)	5 (15)	0.37
Hypertension	4 (24)	12 (55)	16 (41)	0.99
Headache, n (%)	5 (29)	11 (50)	16 (41)	0.33
Primary therapy (i.e. TSS)	8 (47)	6 (27)	14 (36)	0.31

PRL, prolactin (µg/L); IQR, interquartile range; n, numbers; BMI, body mass index; SD, standard deviation

### Characteristics at last follow-up

Patients’ characteristics at the last follow-up are summarized in Table 2. The mean (± SD) follow-up period was 74 ± 48 months, with no differences observed between men with and without ED (*p* = 0.10). Over the long term, PRL levels significantly decreased in both groups, namely from 2628 µg/L (IQR 694–9159 µg/L) to 16 µg/L (IQR 9–30 µg/L) in the non-ED cohort (*p* = 0.01), and from 1831 µg/L (IQR 768–4457 µg/L) to 13 µg/L (IQR 5–24 µg/L) in the ED cohort (*p* = 0.03, Fig. 1). At the last follow-up, serum PRL levels were not significantly different between the two groups (*p* = 0.65). Therefore, remission from hyperprolactinemia was attained in the majority of men (76%); 71% of men in the non-ED cohort and 81% in the ED group (*p* = 0.70), and this was independent of the primary treatment strategy (Surgical: 84% versus medical: 72%, *p* = 0.46). Long-term remission of ED was noted in 16 out of 22 (73%) patients, specifically in all 6 patients in the surgical group compared to 10 (63%) patients in the medical cohort (*p* = 0.13). BMI values decreased over the long term, though not significantly (from 28.4 ± 4.5 kg/m^2^ to 28.0 ± 4.6 kg/m^2^, *p* = 0.17). Likewise, equal changes in patients’ BMI values in the surgically (-0.7 ± 2.9) versus medically (-0.92 ± 3.7) treated patients were noted in the long-term (*p* = 0.86). No significant differences in the BMI values between the two cohorts over the long term were noted (*p* = 0.17, Fig. 1B). A drop in patients' BMI in the ED cohort with persistent ED at the last follow-up was not significantly different from those patients whose ED resolved (-1.8 ± 1.2 kg/m^2^ vs. -1.3 ± 4.3 kg/m^2^, *p* = 0.67). We noted a significant correlation between PRL levels and patients’ BMI at the last follow-up (r = 0.4, *p* = 0.02), but not between baseline values (r = 0.18, *p* = 0.32).

**Table 2:** Patient characteristics at long term follow-up

Characteristics at last follow-up	Non-ED	ED	All patients	P-value
Follow-up time (mts), median (range)	100 (24–174)	46 (24–186)	63 (24–186)	0.1
BMI (kg/m2), mean ± SD	26.9 ± 4.7	27.8 ± 4.4	28.0 ± 4.6	0.17
PRL levels (μg/L), median (IQR)	16 (9–30)	13 (5–24)	13.9 (6–26)	0.65
Affected pituitary axes, n (%)				
Secondary adrenal insufficiency	0 (0)	2 (9)	2 (5)	0.5
Secondary hypothyroidism	0 (0)	0 (0)	0 (0)	1
Gonadotropin deficiency, n (%)	7 (41)	11 (50)	18 (46)	0.75
LH (IU/L), mean ± SD	4.0 ± 3.2	3.5 ± 2.5	3.7 ± 2.8	0.62
FSH (IU/L), mean ± SD	5.3 ± 4.6	4.7 ± 3.6	5.1 ± 4.2	0.72
Testosterone levels (nmol/L) mean ± SD	15.4 ± 7.3	11.6 ± 4.8	12.6 ± 6.2	0.11
Erectile dysfunction, n (%)	1 (6)	6 (27)	7 (18)	0.11
Continuous DA therapy, n (%)	14 (82)	15 (68)	29 (74)	0.46
Headache, n (%)	1 (6)	4 (18)	5 (13)	0.36
Remission, n (%)	12 (71)	17 (81)	29 (76)	0.7

PRL, prolactin (µg/L); IQR, interquartile range; mts, months; n, numbers; BMI, body mass index; SD, standard deviation

**Fig. 2:**
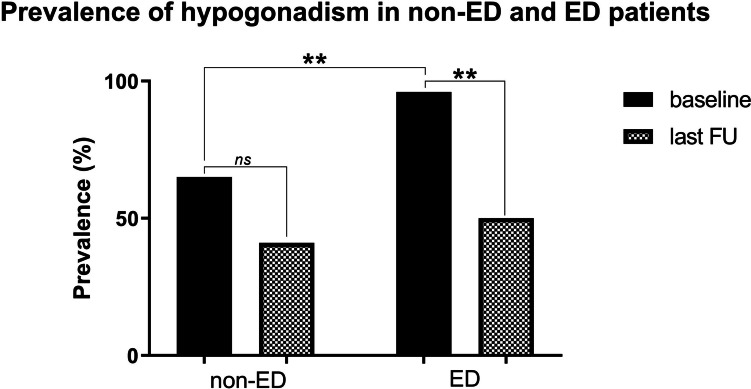
Changes in rates of hypogonadism over time. Compared to baseline, rates of hypogonadism significantly improved in the ED group (96% to 50%, *p* = 0.002), whereas rates in non-ED patients remained stable (65% vs. 41%, *p* = 0.3). There was no significant difference in the prevalence of hypogonadism between ED and non-ED patients at last follow-up (50% vs. 41%, *p* = 0.75), but there was at baseline (96% vs. 65%, *p* = 0.03)

In patients with ED as a presenting symptom, significant remission from ED was noted (56% vs. 18%; *p* = 0.001), as were levels of testosterone (12.1 ± 4.1 vs. 7.3 ± 3.9, *p* = 0.02), rates of hyperprolactinemia (100% vs. 19%, *p* < 0.001), and rates of hypogonadism (96% vs. 50%, *p* = 0.002, Fig. 2). In the non-ED cohort, rates of hypogonadism remained balanced (65% at baseline vs. 41% at the last follow-up, *p* = 0.30), while rates of hyperprolactinemia significantly decreased (100% vs. 29%, *p* < 0.001).

There was no significant difference in the prevalence of hypogonadism in ED versus non-ED patients at the last follow-up (50% vs. 41%, *p* = 0.75), but there were differences at baseline (96% vs. 65%, *p* = 0.03). Testosterone substitution was prescribed in 16 (44%) of all patients, with no difference between both cohorts (ED vs. non-ED; 71% vs. 38%, *p* = 0.20). Additionally, no significant difference in testosterone levels (ED vs. non-ED 11.6 ± 4.8 nmol/l vs. 15.4 ± 7.3 nmol/l, *p* = 0.11) was noted in the long term. Potential risk factors associated with persistent ED over the long term included high baseline BMI levels (OR 1.4, 95% CI 1.0–1.9; *p* = 0.04), whereas the initial adenoma size and the primary treatment strategy (TSS vs. DAs, Table 3) did not show significant associations.

**Table 3:** Risk factors for persistent ED at long-term follow-up

Risk factors for ED at last FU	Univariable Analysis HR (95% CI)	P-value	Multivariable Analysis HR (95% CI)	P-value
Age (years)	1.0 (1.0–1.1)	0.44		
Baseline BMI (kg/m2)	1.4 (1.1–1.9)	**0.02**	1.4 (1.0–1.9)	**0.04**
Cardiovascular risk factors	1.2 (0.1–11.3)	0.85		
Smoking	1.4 (0.2–13.7)	0.76		
Diabetes	1.8 (0.2–18.5)	0.62		
Hypertension	1.1 (0.2–6.7)	0.91		
Baseline PRL levels (μg/L)	1.0 (0.3–3.3)	0.94		
Baseline gonadotropin deficiency	27.9 (0.0–435.2)	0.49		
Testosterone levels (nmol/L)	1.0 (0.8–1.3)	0.84		
Headache	2.6 (0.4–15.4)	**0.3**	2.2 (0.2–25.3)	0.53
Adenoma size (i.e. Macroadenoma)	2.1 (0.3–18.4)	0.49		
Cavernous sinus invasion	1.5 (0.3–8.3)	0.66		
Primary therapy (i.e. DAs)	3.5 (0.0–63.2)	0.35		

BMI, body mass index; CI, confidence intervals; DA, dopamine agonist; HR, hazard ratio; PRL, prolactin

## Discussion

The present study on men with a proven lactotroph adenoma was able to demonstrate, that i) correction of hyperprolactinemia and associated hypogonadism tends to improve ED over the long term (74 ± 48 months) in the majority of men, irrespective of the primary treatment strategy, and ii) in addition to restoring endocrine functions, the observed association between high BMI and persistent ED in men with prolactinomas may suggest the potential benefits of early initiation of weight control programs. However, further research is necessary to establish any definitive relationships.

### ED improvement through control of hyperprolactinemia

Sexuality and reproduction are significant priorities for young patients [1]. A considerable proportion of prolactinoma patients are young, yet men with prolactinomas typically tend to be significantly older than women[4, 5, 7]. This trend may be linked to the fact that men with prolactinomas experience low libido, a symptom that is frequently overlooked or underreported, whereas prolactinoma-associated amenorrhea in women is readily detected and investigated early on [20]. In our cohort, the mean age of 45 years in men with a prolactinoma represents middle adulthood, where there is no increased prevalence of ED in the general population [46]. At diagnosis, the majority of men presented with a macroprolactinoma. When ED becomes evident or is reported by men, they tend to be suffering from longer-lasting hyperprolactinemia and associated hypogonadism. Given their often nonspecific symptoms over a prolonged period, subsequent presentation with larger adenomas is not uncommon.

In general, it is assumed that hypogonadism is one of the main causes of ED[16]. Similarly, in patients with ED and evidence of hyperprolactinemia, testosterone levels might be in the normal range[17], corroborating our results with non-significantly decreased testosterone levels in the ED versus non-ED cohort at the time of diagnosis. Consequently, decreases in prolactin levels due to therapy with DAs have more often been associated with improvement in ED, rather than with increases in testosterone levels[17]. Moreover, a testosterone-independent effect of hyperprolactinemia on ED has been described [50]. Additionally, the role of age in ED among prolactinoma patients is controversial, as the reversal of ED following control of hyperprolactinemia has also been reported in older patients (> 65 years of age)[56]. ED in relation to hyperprolactinemia is not uncommon[17], and thus controlling ED by treating hyperprolactinemia and hypogonadism remains of utmost importance.

Interestingly, Johri et al. found no correlation between baseline PRL levels and patients’ sexual desire or erectile function. However, all patients with severe hyperprolactinemia had an IIEF-5 score < 10 [37]. Thus, measurement of prolactin levels has been suggested in patients with ED until the significance of this potential causality becomes clearer[15]. On the other hand, only a slightly disturbed pattern of ED has been described in hyperprolactinemic men[19]. Nonetheless, systematic measurements of serum PRL revealed low prevalences of marked hyperprolactinemia in ED patients, and intriguingly, very low incidences of pituitary adenomas (0.4%), indicating that other causes of the disease need to be evaluated[16].

### Influence of BMI on ED in prolactinoma patients

Lifestyle changes in obese men with ED have been associated with improvement in sexual function in approximately 30% of cases [31]. Namely, compared to men with a normal BMI, those with a BMI ≥ 29 had about one-third higher risk for ED[14]. In our cohort, the BMI of men with ED was significantly higher than that of men without ED. Generally, weight gain in patients with hyperprolactinemia is not uncommon, yet the exact mechanism remains unclear [29, 55]. Hyperprolactinemia-induced hypogonadism can contribute to obesity[3, 30].

We observed a significant correlation between PRL levels and patients’ BMI in the long term. While there might be a certain age-related increase in body weight over time, hyperprolactinemia has been associated with increased patients’ BMI [11, 30]. Interestingly, weight loss has been observed in patients following DA therapy, suggesting that DA has direct effects on patients’ metabolism [11, 18, 40, 47], with higher doses of DA leading to lower BMI levels[21]. However, the exact mechanism remains unclear, and data are conflicting [33, 58]. While it has been hypothesized that DA improves patients’ BMI, we observed similar changes in the surgical cohort, suggesting that the control of hyperprolactinemia itself, rather than the effect of the DA drugs, accounts for this effect.

Additionally, although longer exposure to hyperprolactinemia might contribute to higher BMI levels in patients with macroprolactinomas[54], there was no difference in the prevalence of macroprolactinomas between the ED and non-ED cohorts, yet ED patients exhibited a significantly higher BMI. This finding is intriguing with an unclear underlying mechanism [32]. It is possible that the normal age-associated increase in body weight also contributes to changes in patients’ BMI, but their influence might be modest in a prolactinoma cohort. As obesity is a modifiable risk factor, early implementation of weight loss interventions may be encouraged in men with prolactinomas. However, while BMI showed an association with persistent ED in this group, the strength of this association remains tentative.

In summary, correction of hyperprolactinemia is crucial, as it often restores erectile dysfunction in many men with prolactinomas. Additionally, weight control might be encouraged in men with prolactinomas, although the association between weight and ED outcomes remains uncertain. Future research directions should explore interactions involving changes in libido during weight control interventions, hyperprolactinemia, and associated hypogonadism. Additionally, investigations should consider potential links with quality of life, impulse control disorders—such as hypersexuality and binge eating—and mood disturbances. These factors all contribute to the holistic care of patients with prolactinomas.

## Conclusion

Long-term correction of hyperprolactinemia and associated hypogonadism restores erectile function in the majority of men with prolactinomas, regardless of the primary treatment strategy. Additionally, early consideration of weight control programs may be beneficial for prolactinoma men with ED, alongside improvements in endocrine deficiencies. However, our findings should be considered preliminary, underscoring the need for larger, more controlled studies to confirm these associations.

## Study limitations

While our study design facilitated the analysis of a homogeneous cohort of prolactinoma patients, encompassing their treatment indications and 20-year follow-up—an essential aspect in evaluating ED—the study is constrained by its sample size and retrospective, single-center design. Therefore, we recognize the limitations posed by our relatively small sample size and the potential for collinearity and confounding in our multivariable analysis.

The standardized 5-item version of the IIEF-5 was not systematically employed to assess ED[52]. Furthermore, by exclusively including patients with severe ED defined as IIEF-5 ≤ 7, our study may have overlooked individuals with milder forms of ED, potentially neglecting those with varying degrees of severity in our analysis.

While BMI was found to be an independent risk factor for ED in the multivariate analysis, caution is necessary because BMI may not autonomously contribute to the risk of persistent ED. There is a possibility that BMI is associated independently of the adjusted variables due to unmeasured confounding, particularly given the small sample size.

Although the follow-up was ≥ 24 months, a longer treatment time might be necessary to assess whether significant changes in body weight occur in some patients. Additionally, the coefficient of variability (CV) was in the range of 3–5%, with assay changes over the many years of data collection of PRL/testosterone/LH/FSH values in patients with lactotroph adenomas, starting from 1996 until December 2015. Furthermore, forms and doses of testosterone replacement therapy in this patient cohort are missing, making quantification of these values not possible. Thus, allocation into groups (i.e., ± testosterone substitution) only reflects a partial aspect of replacement therapy, which may hinder drawing solid conclusions.

## Data Availability

The authors agree to share data upon request.

## Declarations

The study is a retrospective data project that utilized existing data to evaluate the quality of registry data. There was no patient contact for the study, and therefore, no patient consent process was conducted. The Human Research Ethics Committee of Bern (Kantonale Ethikkommission KEK Bern, Bern, Switzerland) approved the project (KEK No. 10–10-2006 and 8–11-2006). The study was conducted in accordance with the ethical standards outlined in the 1964 Declaration of Helsinki and its subsequent amendments.
